# Temporary Membrane Permeabilization via the Pore-Forming Toxin Lysenin

**DOI:** 10.3390/toxins12050343

**Published:** 2020-05-22

**Authors:** Nisha Shrestha, Christopher A. Thomas, Devon Richtsmeier, Andrew Bogard, Rebecca Hermann, Malyk Walker, Gamid Abatchev, Raquel J. Brown, Daniel Fologea

**Affiliations:** 1 Department of Physics, Boise State University, Boise, ID 83725, USA; nisha.shrestha@northwestern.edu (N.S.); christ38@uw.edu (C.A.T.); drichts@uvic.ca (D.R.); andybogard@boisestate.edu (A.B.); malykwalker@u.boisestate.edu (M.W.); gamidabatchev@u.boisestate.edu (G.A.); 2 Biomolecular Sciences Graduate Program, Boise State University, Boise, ID 83725, USA; 3 Department of Biology, Boise State University, Boise, ID 83725, USA; rebecca.hermann@northwestern.edu; 4 Center of Biomedical Excellence in Matrix Biology, Biomolecular Research Center, Boise State University, Boise, ID 83725, USA; rbrown@boisestate.edu

**Keywords:** lysenin, permeabilization, pore forming toxins, chitosan

## Abstract

Pore-forming toxins are alluring tools for delivering biologically-active, impermeable cargoes to intracellular environments by introducing large conductance pathways into cell membranes. However, the lack of regulation often leads to the dissipation of electrical and chemical gradients, which might significantly affect the viability of cells under scrutiny. To mitigate these problems, we explored the use of lysenin channels to reversibly control the barrier function of natural and artificial lipid membrane systems by controlling the lysenin’s transport properties. We employed artificial membranes and electrophysiology measurements in order to identify the influence of labels and media on the lysenin channel’s conductance. Two cell culture models: Jurkat cells in suspension and adherent ATDC5 cells were utilized to demonstrate that lysenin channels may provide temporary cytosol access to membrane non-permeant propidium iodide and phalloidin. Permeability and cell viability were assessed by fluorescence spectroscopy and microscopy. Membrane resealing by chitosan or specific media addition proved to be an effective way of maintaining cellular viability. In addition, we loaded non-permeant dyes into liposomes via lysenin channels by controlling their conducting state with multivalent metal cations. The improved control over membrane permeability might prove fruitful for a large variety of biological or biomedical applications that require only temporary, non-destructive access to the inner environment enclosed by natural and artificial membranes.

## 1. Introduction

The ability to intracellularly deliver ions, nucleic acids, peptides, proteins, drugs, activators, inhibitors, and labeled compounds that interact with specific targets or modulate essential biological functionalities has enabled unprecedented advances in science, biotechnology, and medicine. The non-permeant nature of the plasma membrane, which often acts as a barrier that prevents access to the cytosol, frequently challenges the deployment of solutes to intracellular environments. For well over a century, investigations focused on introducing non-permeant cargoes into cells while preventing irreversible damage or unintentional perturbations of cellular functionalities led to numerous strategies for mediating intracellular transport [1,2,3,4,5,6,7,8,9,10,11,12,13]. With this goal in mind, gaining access to the cytosol by using pore-forming proteins (PFPs) that disrupt the membrane barrier function and introduce conductive pathways that facilitate a diffusion-driven transport of solutes across membranes is a promising new mechanism [8,9,10,14,15]. In addition to allowing the passage of relatively large molecules [8,9,10,15,16], the potential modulation of the membrane permeability through intrinsic regulatory mechanisms might provide unique controls of the barrier function previously difficult to attain by alternative techniques. As an example, ion channels present high transport rate, selectivity, and regulation [17,18,19]; with a few notable exceptions [2], their narrow opening and high selectivity limit their applicability to intracellular delivery of specific, small-sized ions and compounds. Scientists have utilized pore-forming toxins (PFTs) as alternative pathways for intracellular delivery, which generally have a larger diameter and may allow passage of larger molecules, to address this shortcoming [8,9,10,14,16,20]. A concerning problem that is associated with the use of PFTs is a poor control over their conducting state, which might prove fatal for cells that are unable to engage internal repair mechanisms. Ultimately, such problems may lead to uncontrolled leakage, the dissipation of electrochemical gradients, and cellular death [21,22,23,24,25,26,27]. A widely used PFT for intracellular delivery purposes is Streptolysin O (SLO), a cholesterol-dependent cytolysin [8,9,10,16,28,29]. SLO is not endowed with clear regulatory mechanisms [30]; nonetheless, extensive purification followed by titration and further initiation of membrane repair mechanisms by Ca^2+^ addition allows for the delivery of large molecules while maintaining excellent cellular viability [8,9,10,16]. The recovery protocols vary greatly among reports, and careful optimization is recommended before application to any cell line [9]. Furthermore, this approach might not be applicable at all to artificial membrane systems, which lack internal repair systems. As an improvement with regard to endowing PFTs with regulatory mechanisms, the alpha-hemolysin channel was successfully engineered to function as a reversible switch that is responsive to Zn^2+^ ions [7,31], yet channel reopening might unintentionally occur if Zn^2+^ is depleted, and the rather narrow diameter of the conductive pathway might limit the passage of solutes larger than ~1 kDa in size [7].

Although it is recognized that achieving better control of the channels’ conducting state has the potential to improve PFT’s applicability to intracellular delivery, in most cases opening and closing conducting pathways in the cell membrane in a safe, robust, and well-controlled manner has yet to be achieved. We propose using lysenin channels as nano-valves that control the permeability of natural and artificial lipid membranes and enable free passage of non-permeant molecules to mitigate the challenge. Lysenin, a PFT that is found in the coelomic fluid of *E. fetida*, oligomerizes and introduces large nonameric channels (~3 nm diameter) in artificial and natural cell membranes containing sphingomyelin [32,33,34,35,36], a major component of mammalian cell membranes. The large channel enables the diffusion of non-permeant molecules that cannot pass through the narrow opening of other PFPs. In contrast to many other toxins, lysenin channels possess unique, intrinsic regulatory features [33,37,38] that make it a good candidate for intracellular delivery. For example, sub-micromolar concentration of trivalent metal cations closes the channel through a reversible ligand-gated mechanism [39,40]. Trivalent metal ions bind to a specific site on the lumen and induce conformational transitions from open to closed state, hence blocking the channel, as observed in prior experiments that employed single channel investigations [39,40]. Interestingly, divalent metals and more voluminous trivalent ions elicit conformational changes that result in the channels adopting a sub-conducting state (half-open) [39,40]. In most cases, multivalent ion removal by chelation or precipitation reinstates the original macroscopic conductance of lysenin channels reconstituted in artificial membrane systems [39,40]. In contrast, cationic polymers, such as polyethylenimine or chitosan, irreversibly block the channels and obliterate the membrane’s macroscopic conductance through a mechanism that might employ induced-gating and polymer trapping into the channel’s lumen [41].

This work demonstrates that lysenin channels may be used to control the permeability of both natural and artificial membranes by modulating their conductance with chitosan, cell culture media, or multivalent metal ions. We examined both suspended and adherent live cells, and artificial membrane systems (planar lipid bilayers and liposomes) in electrophysiology, microscopy, and spectroscopy assessments to explore this hypothesis. Our results suggest that this loading methodology, with the quick and well-controlled resealing of the permeabilized membranes, is applicable to cells and artificial lipid membrane systems, and ensures excellent post-treatment cell viability.

## 2. Results and Discussion

### 2.1. Investigations of the Effect of Indicators and Culture Media on the Conductance of Lysenin Channels Reconstituted in Artificial Membranes

**Figure 1:**
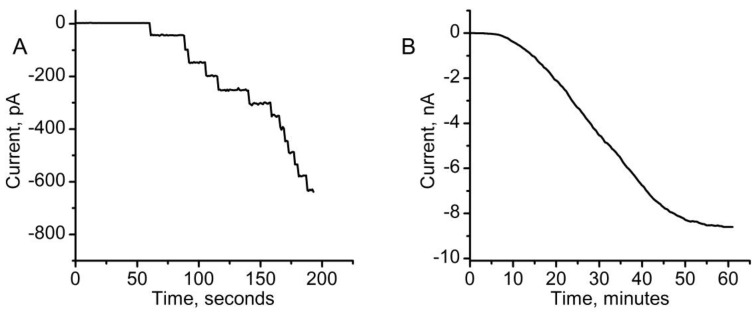
Lysenin channels reconstitution in artificial lipid membranes. (**A**) Shortly after lysenin addition, the ionic currents presented a discrete, step-wise, and uniform variation, indicative of individual channel insertion. (**B**) The macroscopic current reached a steady-state in ~1 h, indicative of insertion completion. Each plot represents a typical trace representative for the given experimental conditions.

Our explorations on the temporary permeabilization of membranes via lysenin channels inserted into the membrane of live cells rely on dyes and media more complex than the simple ionic solutions that are traditionally used for assessments performed in artificial membrane systems. Multivalent cations significantly inhibit the macroscopic conductance of lysenin channels [39,40], which affect their capability to create transmembrane conductive pathways that provide access to the cytosol. Therefore, preliminary testing of the effects of media and dyes on lysenin’s conductance in artificial membrane systems was performed to identify potential interferences with permeabilization. The reconstitution of lysenin channels in planar bilayer lipid membranes in buffered KCl solutions revealed the uniform, step-wise change of the ionic currents, indicative of individual channel insertion [33,40] (Figure 1A). The completion of the insertion process was indicated by the steady state of the ionic current that was achieved in ~1 h (Figure 1B), after which the lysenin still free in solution was removed by buffer exchange and the test solutions were added to the ground reservoir (to simulate the extracellular environment).

**Figure 2:**
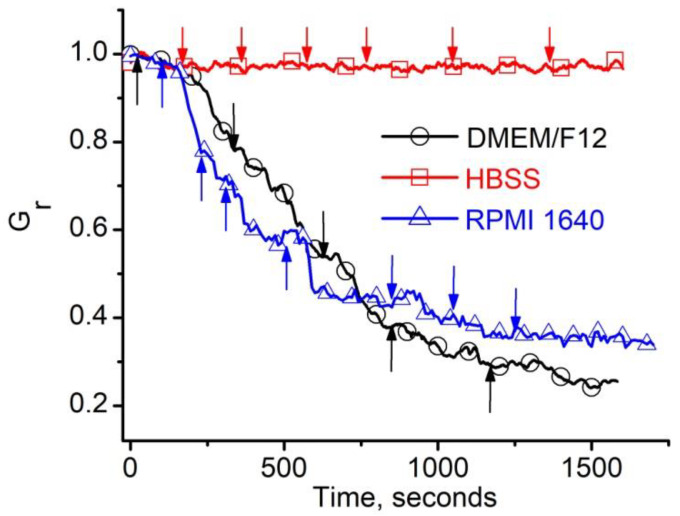
Effects of culture media on the macroscopic conductance of lysenin channels. When the electrolyte buffer was exchanged with DMEM/F12 (50 µL/step) or RPMI 1640 (100 µL/step), the relative conductance of the membrane decreased significantly, indicating a strong inhibitory effect. In contrast, similar addition of HBSS elicited only minor, negligible changes in the macroscopic conductance. Media exchange is indicated by arrows. The plots are constructed by using experimental ionic current data recorded from typical traces, and the symbols were added for guidance.

We recorded the ionic currents upon successively replacing small amounts of the buffered KCl solution in the ground reservoir with the above media (50–100 µL/step) to assess the influence of the Hanks’ Balanced Salt Solution (HBSS) and cell culture media commonly used for culturing ATDC5 and Jurkat cells (DMEM/F12, and RPMI 1640—see the Materials and Methods section for detailed descriptions) on the macroscopic conductance of lysenin channels. The ionic currents were converted into relative macroscopic conductance (G_r_) to facilitate a comparison between experiments comprising a different number of inserted channels. Each step of replacement with DMEM/F12 elicited a significant decrease in the G_r_ (Figure 2); in contrast, negligible changes in conductance were observed upon successive replacement with HBSS (Figure 2). Similar to DMEM/F12, a conductance decrease was also observed after replacing small amounts of buffered KCl with RPMI 1640 (Figure 2). Such inhibitory effects may be a result of the complex compositions, which include multiple charged species that affected the conductance of lysenin channels. HBSS has a much simpler composition, and multivalent charged species that would potentially affect the channels’ conductance are absent. Therefore, we assumed that DMEM/F12 or RPMI 1640 can be further used to reinstate the barrier function of a membrane permeabilized with lysenin channels, while HBSS would be suitable for achieving effective membrane permeabilization under the provision that this medium does not affect the channel insertion itself.

**Figure 3:**
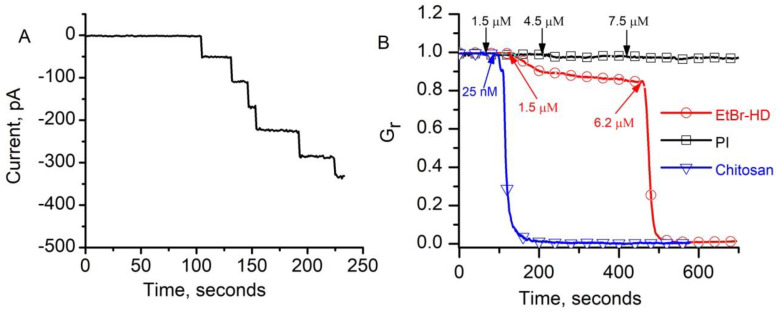
Effects of intercalating agents and chitosan on the macroscopic conductance of lysenin channels reconstituted in planar lipid membranes with Hanks’ Balanced Salt Solution (HBSS) as support electrolyte. (**A**) The HBSS bulk electrolyte did not affect channel insertion. (**B**) EtBr-HD addition inhibited the macroscopic conductance of lysenin channels. In contrast, no major changes in macroscopic conductance were observed upon successive addition of Propidium Iodide (PI). The lysenin-permeabilized membrane bathed by HBSS and PI underwent resealing after addition of chitosan. Each graph represents a typical trace of data recorded in the given experimental conditions. All the data points in panel (**B**) are experimental values, and the symbols have been added to facilitate identification. The arrows indicate addition of the dyes and chitosan, and their concentrations.

We asked whether HBSS would be the choice for inserting conducting lysenin channels into the membranes of live cells since DMEM/F12 and RPMI 1640 proved unsuitable for permeabilization experiments. We reconstituted lysenin channels in planar bilayer lipid membranes exposed to HBSS to test the potential influence of HBSS on channel insertion. Individual channel insertion was monitored from the variation of the ionic currents that were recorded at -80 mV bias potential (Figure 3A). The uniform, stepwise variation of the current resembled the insertion of lysenin channels in planar membranes exposed to buffered KCl solutions (as shown in Figure 1A) [33,34,40], leading to the conclusion that HBSS did not affect the insertion process. Additionally, one might observe that no ionic current was recorded at the beginning of the trace, which indicated that no changes in the membrane permeability occurred when the membrane was exposed to HBSS. Next, we extended the investigations to include the intercalating agents proposed for use as indicators of membrane permeability, i.e., Ethidium Bromide homodimer (EtBr-HD) and Propidium Iodide (PI), which were added as small aliquots to the ground side of the membrane. The first addition of EtBr-HD (final concentration 1.5 µM) induced a visible decrease of the ionic current through the lysenin-permeabilized membrane, which was indicative of a diminished macroscopic conductance (Figure 3B). When the bulk concentration of EtBr-HD was raised to 6.2 µM, the ionic current quickly decreased to near zero. A possible explanation for this undesired effect might be provided from the structural analysis of the dye. EtBr-HD is a relatively long molecule that bears four positive charges that are provided by the included amine groups. These positive charges may induce reversible or irreversible conductance changes by either gating [40] or a gating and trapping mechanism [41]. Although we did not investigate this hypothesis any further, we concluded that EtBr-HD is not suitable for use in experiments that are aimed at inducing controlled permeability via lysenin channels. 

We similarly tested PI for this purpose, which has a much smaller positive charge than the EtBr-HD, therefore a weaker interaction with the lysenin channels and a lower impact on the macroscopic conductance was expected. The addition of PI to the ground reservoir (1.5 µM final concentration) induced negligible changes in the open current, which suggests that PI does not modulate the channel’s conductance (Figure 3B). This observation was supported by a further increase of the PI concentration in the bulk (i.e., 4.5 µM and 7.5 µM), which also elicited insignificant changes of the macroscopic currents and confirmed PI as a suitable candidate for lysenin-induced permeability experiments. In the same line of preliminary explorations, we asked whether the PI in the bulk solutions might prevent channel blockage that is elicited in the presence of chitosan [41], intended to be added as an irreversible channel blocker. Our results (Figure 3B) show that chitosan addition (25 nM final concentration) to the HBSS bulk in the presence of 6 µM PI annihilated the macroscopic conductance of lysenin channels inserted into the planar lipid membrane. This result supported the feasibility of the proposed approach for obtaining controlled membrane permeability by irreversibly controlling the lysenin’s conducting state with chitosan.

Working concentrations of calcein-AM and Alexa Fluor 546 Phalloidin (AF546-Phal) were also tested in similar electrophysiology experiments; none of them elicited a major influence on the lysenin channels’ macroscopic conductance, and their effects were similar to the HBSS (as shown in Figure 2).

All of the data presented in Figure 1, Figure 2 and Figure 3 show typical traces from individual experiments. Nonetheless, numerous independent experiments performed in identical experimental conditions qualitatively and quantitatively replicated the presented results. The channel insertion process is a statistical one; therefore, average traces for the insertion events cannot be provided since the insertions occur at random times. In the same line, the experimental setup made it difficult to replicate the addition of media and dyes at identical time intervals for parallel experiments. Although all of the samples showed similar evolutions of the ionic currents, the time evolution of the changes differed between the experiments. This might be easily explained by taking into account that the mixing in the chambers was not uniform and noise considerations often required adjustments of the rotational speed of the stir bars in the chambers, therefore influencing the mixing. In spite of these shortcomings, the relative changes in macroscopic conductance that were observed for identical conditions and after achieving steady state were similar in parallel experiments.

### 2.2. Investigations on Jurkat Cells: Viability and Permeabilization Assays

#### 2.2.1. Viability Assessments

**Figure 4:**
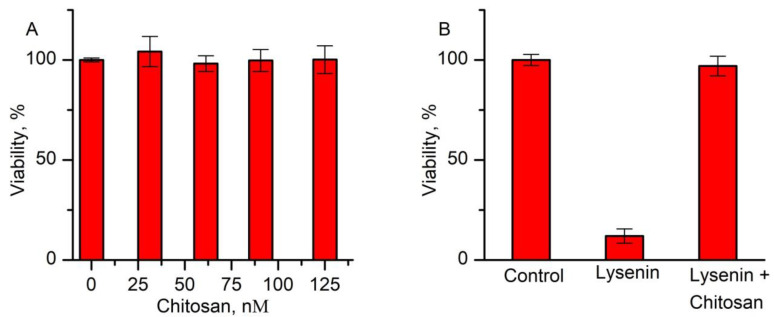
Viability tests on Jurkat cells exposed to chitosan and lysenin. (**A**) Exposure of Jurkat cells to chitosan concentrations up to 125 nM elicited only minor, negligible changes in cellular viability. (**B**) Jurkat cells exposed to only lysenin (10 nM final concentration) suffered massive damage, leading to a reduced viability. Chitosan addition (25 nM final concentration) 20 min. after lysenin exposure protected the cells and led to almost complete recovery. The experimental data represents averages of three independent experiments (n = 3, ±SD).

Our next explorations focused on testing the cytotoxic effects of chitosan, lysenin, and their combination on the viability of Jurkat cells (Figure 4) by comparison with negative controls (samples that were incubated without lysenin and/or chitosan). Although chitosan is considered to be a bio-inert molecule, there is not sufficient information in regard to its potential toxicity manifested against Jurkat cells. The Alamar Blue (resazurin) assay test [42] that was performed after 20 h incubation of Jurkat cells with increasing concentrations of chitosan showed no significant changes in the cellular viability (Figure 4A), even at five times (up to 125 nM) the concentration needed to block lysenin channels. 

We performed the resazurin viability test by employing 10 nM lysenin and 25 nM chitosan added after 20 min. exposure to lysenin alone to assess the influence of lysenin channels on the viability of Jurkat cells with and without chitosan-induced channel blockage (Figure 4B). This test did not include PI addition, which might also affect the cellular viability. Exposure to lysenin alone led to a significantly lower viability, estimated at ~12% by comparison to the negative control. However, the cells that were treated with chitosan after lysenin-induced permeabilization did not suffer major damage, as inferred from the high viability rate (~97%). This result suggests that the addition of chitosan prevented extensive membrane damage induced by lysenin and enabled remarkable recovery.

#### 2.2.2. Achievement of Controlled Permeability of Jurkat Cell Membranes

**Figure 5:**
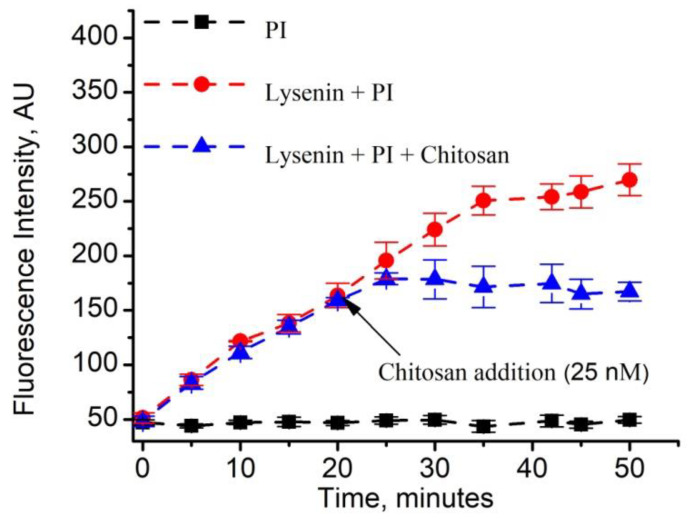
Chitosan controls the lysenin-mediated transport of PI across the membrane of Jurkat cells. PI did not cross the cell membrane in the absence of lysenin, as indicated by the steady fluorescence intensity. In contrast, a continual increase in fluorescence, indicative of PI intercalation, was observed after lysenin addition (10 nM final concentration). For a similarly permeabilized sample, addition of chitosan 20 min. after the initiation of permeabilization quickly stabilized the fluorescent intensity and indicated that PI was prevented from further crossing the cell membrane. The symbols represent average values from four experiments (n = 4, ±SD) that comprised individual samples made from the same cell culture batch.

Fluorescence spectroscopy was employed to monitor the cells by specifically tracking the uptake of dye into the nucleus of cells exposed to PI alone, PI and lysenin, and PI and lysenin subsequently blocked with chitosan in order demonstrate that lysenin and chitosan enable the achievement of controlled permeability of Jurkat cells membranes (Figure 5). Upon continual exposure to PI only, the cells presented a steady, weak fluorescence for the entire duration of the experiment (~50 min.), suggesting that PI did not accumulate into the nucleus. The lack of intercalation suggests that PI did not cross the plasma membrane, which would be indicated by an increasing fluorescence signal. In contrast, the addition of lysenin (10 nM) in the presence of PI (1.5 µM) showed a gradual increase of fluorescence, which seemed to asymptotically reach saturation, thus strongly suggesting that PI crossed the membrane and intercalated into the nuclear DNA for the entire duration of the experiment (Figure 5). The samples containing PI and lysenin channels in the membrane showed a very similar pattern until the 20 min. time mark, when chitosan (25 nM final concentration) was added to induce a quick blockage of lysenin channels.

The fluorescence intensity that was recorded a few minutes after chitosan addition (~25 min. from the beginning of the experiment) showed a decreased value when compared to the samples that included lysenin, but no added chitosan. This difference accentuated over time and the samples that were blocked with chitosan showed a rather stable, steady state of the fluorescence signal. The slight increase that was immediately observed after chitosan addition was interpreted as originating from PI that was still in the cytosol (though not yet intercalated into the nuclear DNA before channel blockage) or passed at a lower rate into the cytosol before complete membrane resealing. The relatively steady fluorescence signal that was recorded after chitosan addition suggests that the barrier function of the membrane was reinstated. This also confirms that no major changes in cell viability occurred after chitosan addition, which would lead to a gradual increase of the fluorescence intensity, owing to PI’s diffusion through the compromised membranes of dead cells after channel blockage.

### 2.3. Investigations on ATDC5 Cells: Permeabilization Assay

#### Investigations on ATDC5 Cells

**Figure 6:**
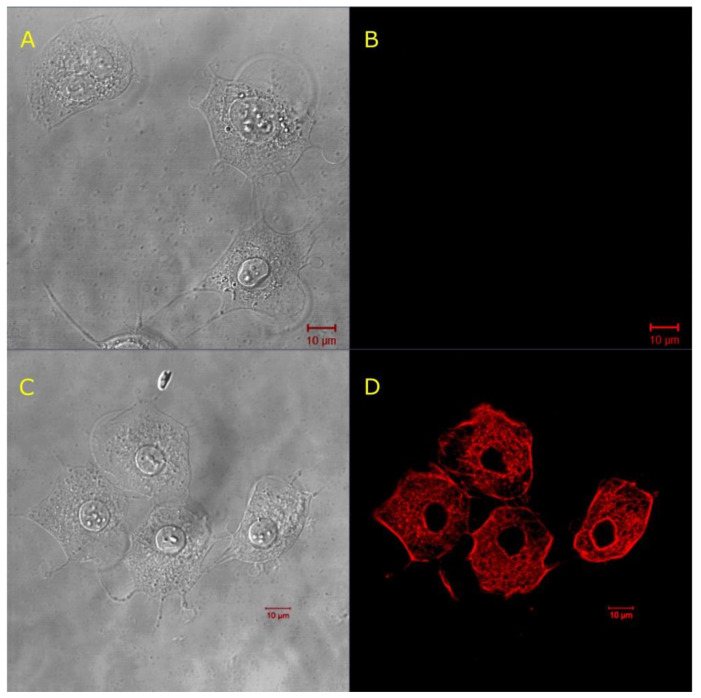
Channels enable the transport of non-permeant AF546-Phal across the membrane of adherent ATDC5 cells. (**A**,**C**) Transmitted light microscopy imaging shows the presence of cells. (**B**) Cells not exposed to lysenin lack the red fluorescence, suggesting that AF546-Phal did not cross the membrane of non-permeabilized cells. (**D**) AF546-Phal was detected in the cytosol of cells exposed to lysenin (10 nM final concentration).

Our next investigations aimed at using lysenin channels to control the permeability of ATDC5 cell membranes to phalloidin, a bicyclic heptapeptide toxin that is extracted from the mushroom *Amanita phalloides* [43]. Phalloidin binds strongly to the polymeric, filamentous actin (F-actin), hence preventing its further depolymerization [44,45,46]. Phalloidin is freely permeant through the membrane of hepatocytes, but, for other cells, access to the cytosol must be provided by alternative approaches, such as microinjection, optoporation, electroporation, or others [1,9,16,47,48]. Therefore, we explored the ability of lysenin channels to open conductance pathways that facilitate phalloidin’s passage into the cytosol of adherent ATDC5 cells. We used the fluorescent AF546-Phal conjugate as an indicator for transmembrane transport through lysenin channels to enable microscopy imaging. Our experiments comprised adherent ATDC5 cells that were exposed to lysenin and phalloidin conjugate as well as control samples that excluded the use of lysenin. After cell identification by microscopy under transmitted light (Figure 6A,C), the presence of fluorescent, intracellular phalloidin was identified by confocal microscopy.

**Figure 7:**
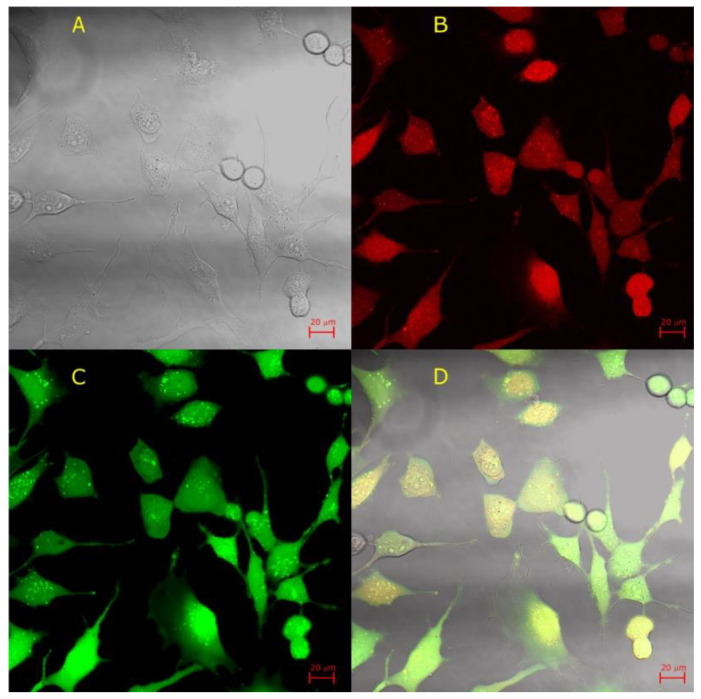
Cells loaded with phalloidin via lysenin channels remain viable after over five hours incubation in DMEM media. (**A**) Transmitted-light image of ATDC cells upon exposure to lysenin, phalloidin, and calcein-AM. (**B**) AF546-Phal was detected inside the lysenin-permeabilized ATDC5 cells (red channel). (**C**) The lysenin-permeabilized cells further exposed to inhibitory DMEM/F12 remained viable, as suggested by the green fluorescence presented by calcein-AM cleaved by esterases in the cytosol (green channel). (**D**) Overlap of transmitted, red, and green channel images.

The cells that were exposed to AF546-Phal in the absence of lysenin showed no red fluorescence (Figure 6B). In contrast, the cells exposed to both AF546-Phal and lysenin presented a strong fluorescent signal (Figure 6D), which was indicative of binding to F-actin after crossing the cell membrane. We concluded that the intact cell membranes constituted a barrier for the passage of the fluorescent phalloidin conjugate, and lysenin addition was essential for rendering the cell membranes permeable to the heptapeptide. However, these experiments were performed at short time scales, and the viability of the cells after treatment was not assessed. Lysenin and phalloidin both exert cytotoxic effects by either dissipating the electrochemical gradients across the cell membranes or inhibiting the depolymerization of F-actin, respectively. We added calcein-AM as a live-cell indicator to examine the cellular viability after treatment with lysenin and AF546-Phal [49]. The cells were exposed to AF546-Phal and lysenin similarly to the previous experiments described in this section. After 10 min. of incubation with lysenin and AF546-Phal, the samples were washed twice, and then incubated in DMEM/F12 (which also acted as channel blocker) for over five hours. The confocal microscopy analysis (Figure 7) of the cells that were treated with lysenin, AF546-Phal, and then blocked with DMEM/F12 was performed after the addition of calcein-AM as a viability indicator and subsequent washing. Image analysis showed the presence of lysenin-permeabilized cells (Figure 7A), which also presented a strong red fluorescence (Figure 7B), suggesting that lysenin channels altered the barrier function of the membrane and allowed cytosol access. The same samples showed the specific green fluorescence of the calcein dye that was released from the AM-conjugate by the catalytic activity of esterases (Figure 7C), hence indicating the cellular viability of the lysenin-exposed cells. The dye distribution inside cells is presented in the overlap image (Figure 7D).

**Figure 8:**
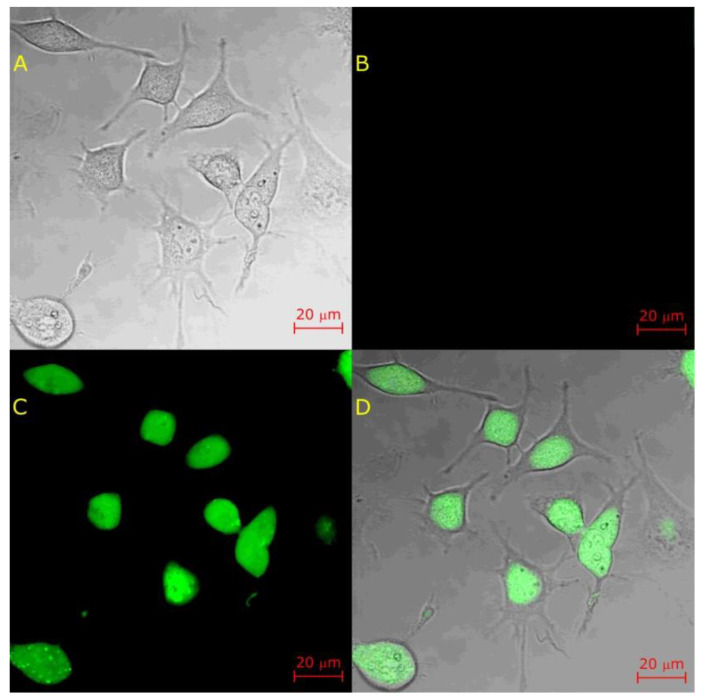
AF546-Phal does not cross the membranes of ATDC5 cells in the absence of lysenin. (**A**) Transmitted light image of cells exposed to AF546-Phal and calcein-AM. (**B**) The lack of red fluorescence indicates that AF546-Phal did not cross the membranes not exposed to lysenin. (**C**) The strong green fluorescence recorded after over five hours indicates that the cells remained viable. (**D**) The overlap of transmitted and green channel images shows calcein distribution inside cells.

We prepared identical cell samples that underwent an otherwise identical procedure but excluded lysenin addition to demonstrate that lysenin is required to achieve permeabilization at longer time scales. The cells that were identified under transmitted light (Figure 8A) showed no red fluorescence (Figure 8B), indicating that lysenin addition is essential for the AF546-Phal to cross the membranes. However, strong fluorescence was observed for calcein (Figure 8C), which was relatively uniformly distributed into the cytosol (Figure 8D).

### 2.4. Reversible Liposome Loading and Unloading via Lysenin Channels

**Figure 9:**
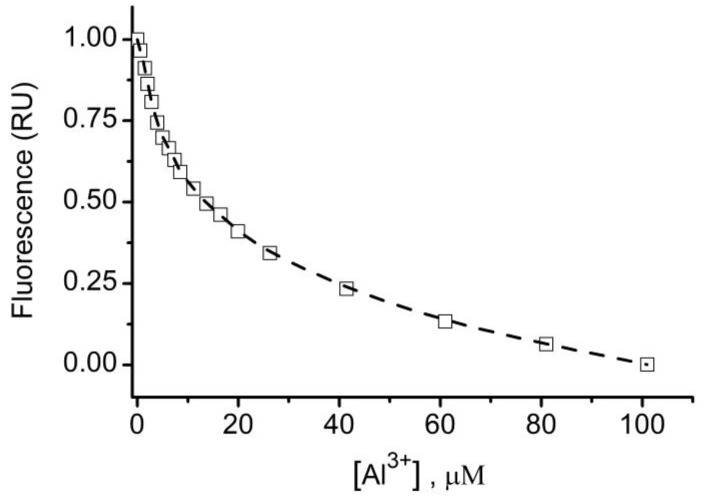
Calcein fluorescence is quenched by Al^3+^ ions in a concentration dependent manner. The relative fluorescence of 50 µM calcein prepared in 135 mM KCl and 20 mM HEPES (pH 7.2) decreased significantly upon Al^3+^ addition; 100 µM cation concentration effectively quenched the calcein’s fluorescence.

We conducted experiments using liposomes to demonstrate that lysenin channels reconstituted into artificial membranes may facilitate entrapment and release of non-permeant molecules. We tracked the transmembrane transport of non-permeant calcein to assess loading into and release from liposomes by microscopy. Additionally, Al^3+^ cations were utilized in these experiments given their ability to quickly and reversibly modulate lysenin channel’s conductance by a ligand-gating mechanism [39]. It is worth noting that this approach provided an opportunity to simplify the procedure of assessing lysenin-mediated loading. While unincorporated calcein may be removed by classic separation techniques such as dialysis, chromatography, or centrifugation, we exploited a distinctive property of calcein, i.e., the fluorescence quenching by multivalent metal ions [50]. This property enabled the elimination of the bulk fluorescence to render the loaded liposomes fluorescent against the background. While a previous report shows that Al^3+^ is among the most potent multivalent ion for the inhibition of the macroscopic conductance of lysenin [39], no consistent reports have determined the fluorescence quenching effects of Al^3+^ on calcein. Our fluorescence spectroscopy exploration indicated that successive addition of Al^3+^ cations to a 50 µM calcein solution strongly quenched the fluorescence in a concentration-dependent manner (Figure 9), and that 100 µM Al^3+^ reduced the calcein fluorescence to negligible values. In addition, fluorescence quenching occurred at concentrations larger than what is needed to efficiently inhibit the macroscopic conductance of lysenin channels [39]. The present work exploits this feature for reducing the background fluorescence by adding the quenching multivalent cations to the liposome mixture, assuming that these ions would rapidly close the channels and only quench the calcein in the external solution. Once the channels were blocked, it was anticipated that the sealed membrane would prevent cation access to the inner environment and protect the entrapped dye from quenching.

**Figure 10:**
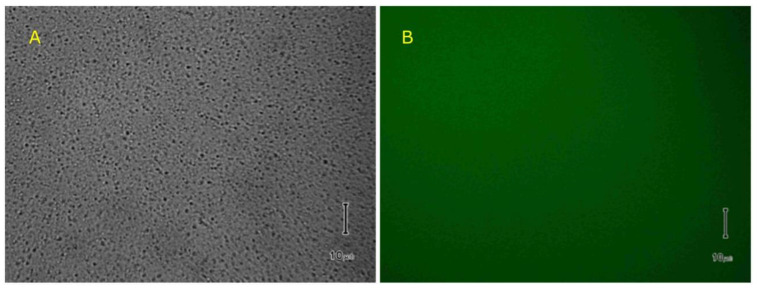
Experiments conducted on liposomes. (**A**) Transmitted light imaging taken after lysenin and calcein addition show intact, non-aggregated liposomes. (**B**) No fluorescent liposomes have been identified against the background after calcein addition to the solution of liposomes containing open lysenin channels in the membrane. Scale bar = 10 µm.

Spherical membrane permeabilization was performed by mixing 50 µL liposomes with 1 µL of 30 nM lysenin at room temperature. After the addition of calcein (50 µM final concentration) to the solution containing permeabilized liposomes, the sample was left to equilibrate for six hours at room temperature in a dark container. Wide-field transmitted light microscopy imaging (Figure 10A) indicated the presence of uniform and well dispersed liposomes in the sample solution. The same sample that was analyzed by fluorescence microscopy did not show any individual liposomes against the background (Figure 10B) and we concluded that the fluorescent dye did not accumulate either on their surfaces or inside their membranes.

**Figure 11:**
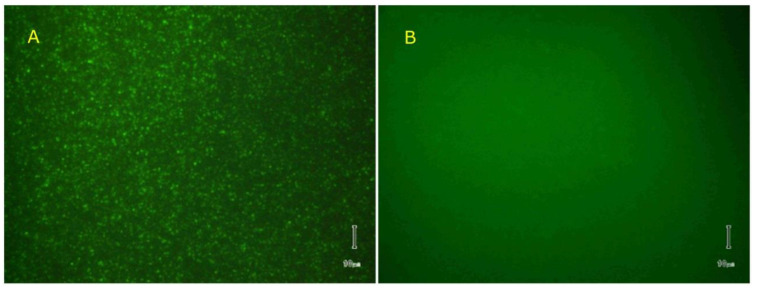
Al^3+^ ions reversibly control the lysenin-induced permeability in liposome membranes. (**A**) Addition of Al^3+^ (100 µM final concentration) blocked the lysenin’s conducting pathway, trapped the calcein inside, quenched the non-entrapped calcein and rendered the liposomes fluorescent against the background. (**B**) Addition of 10 mM phosphate buffer led to Al^3+^ precipitation, channel reopening, and a more homogeneous distribution of the dye inside and outside of liposomes. Scale bar = 10 µm.

The open channels in the liposome membranes were blocked by the addition of 100 µM Al^3+^ to the mixture, which obliterated the transport through the lysenin channels, trapped the calcein inside liposomes, and quenched the non-loaded dye. The fluorescence microscopy images were taken one hour after Al^3+^ addition and revealed the presence of highly-fluorescent liposomes, indicative of successful loading and membrane resealing (Figure 11A). We also observed that not all the liposomes incorporated the dye by switching the illumination from fluorescence to visible-transmitted light conditions. Although such an effect could be a result of incomplete membrane resealing by channel blockage upon Al^3+^ addition and calcein leakage, the most reasonable explanation relies on the fact that not all of the large liposomes produced by extrusion were unilamellar. Lysenin might interact with multilamellar structures, but it cannot be assumed that the inserted pores will completely penetrate thick, multilayered structures to create a conducting pathway between the inner water-filled cavity and the external solution. We precipitated the Al^3+^ cations with phosphate [39] (10 mM final concentration) added directly to the liposome solution to release the incorporated calcein. The fluorescence image taken 20 min. after phosphate-induced precipitation of the trivalent cations showed no fluorescent liposomes (Figure 11B). However, transmitted-light imaging showed intact liposomes, like those in Figure 10A, confirming dye leakage through the lysenin channels that reopened after phosphate-removal of the conductance-inhibiting Al^3+^ cations.

## 3. Conclusions

Our work provides evidence that lysenin channels can mediate the controlled transport of non-permeant ions and molecules in both natural and artificial membrane systems. Lysenin channels work as controlled transmembrane nano-valves for both suspended and attached cells. This ability to create conducting pathways that can be further occluded by bio-inert cationic polymers, such as chitosan, is essential in maintaining the high viability of cells undergoing temporary permeabilization. Lysenin is reported as presenting weak cation selectivity [34], which might potentially influence the transport of cationic solutes. However, this was not the case for any of the employed dyes and labels, although it might manifest for other compounds. Genetic engineering might adjust the channel’s selectivity, but such future experiments must ensure that the regulatory features, which are essential for permeability control, are preserved. Controlling the transport in and out of liposomes might further provide novel avenues for the design and development of liposome-based nano-carriers capable of delivering their cargo upon exposure to physical and chemical stimuli. While such applications may be easily developed for in vitro experiments, careful consideration must be taken with regards to the potential toxicity elicited by lysenin for in vivo applications. We showed that trivalent metal ions and cationic polymers block the lysenin channels, while other natural compounds may prevent lysenin binding to membranes and/or oligomer formation [51]; given the complexity of in vivo systems, other molecules may interfere with the channel’s transport properties. The limited size of the lysenin channel opening in comparison to other toxins (i.e., SLO [16]) might potentially restrict the proposed methodology. Nonetheless, ions, dyes, small peptides, and even single stranded nucleic acid molecules diffuse through the open lysenin channel [32,52], which is anticipated to lead to numerous scientific, biotechnological, and biomedical applications that are based on this controlled permeabilization approach.

## 4. Materials and Methods 

### 4.1. Bilayer Lipid Membrane Experiments 

Planar bilayer lipid membranes were formed from asolectin (Aso, Sigma–Aldrich, St. Louis, MO, USA), cholesterol (Chol, Sigma–Aldrich), and sphingomyelin (SM, Avanti Polar Lipids, Alabaster, AL, USA) dissolved in n-decane (Fisher Scientific, Hampton, NH, USA) at a weight ratio 10:5:4 with a final concentration of 10 mg/mL Aso in the solvent. The membranes were produced in a custom-made chamber while using the painting method [37,38] and the two reservoirs filled with either working electrolyte (135 mM KCl, 20 mM HEPES, pH 7.2) or other solutions as described in the main text. A 0.1 mg/mL stock solution of lysenin (Sigma–Aldrich) was obtained by solubilizing the lyophilized monomer in working electrolyte, which was further diluted as needed for the experiments. The bilayer membrane formation and its integrity were assessed by capacitance and conductance measurements that were aided by two Ag/AgCl electrodes embedded in the two reservoirs and wired to an Axopatch 200B amplifier (Molecular Devices, San Jose, CA, USA) that fed a Digidata 1440A digitizer for signal visualization and recording with the pClamp 10 software (Molecular Devices). The ionic currents were monitored and recorded at −80 mV bias potential (to avoid the voltage-induced gating that manifests at positive voltages [37,38]), a sampling rate of one sample/s, a 1 kHz hardware filter, and a 0.1 kHz software filter. The solutions in the chamber were stirred at room temperature with a low noise magnetic stirrer (SPIN-2 bilayer stirplate, Warner Instruments, Hamden, CT, USA).

After the bilayer membrane was formed and stabilized, lysenin channel insertion was initiated by addition of lysenin into the ground reservoir under continuous stirring. In most cases, a lysenin concentration of 1 pM sufficed for achieving a reasonable ionic current (in the nA range). The concentration of the lysenin in the reservoir was increased up to 100 pM by successive additions when needed. Once a stable population of inserted channels was achieved, the buffer was exchanged to remove the free lysenin and changes in ionic currents induced by addition of culture media or dyes were recorded. AF546-Phal, EtBr-HD, PI, calcein-AM, and calcein were purchased from ThermoFisher Scientific (Waltham, MA, USA). Medium molecular weight chitosan (200,000 average MW, Fisher Scientific, Pittsburgh, PA, USA) was prepared as a stock solution of 1% (*w/v*) in 0.1 M acetic acid (Sigma–Aldrich) and further diluted with deionized (DI) water. All the other common chemicals were purchased from various distributors and prepared in accordance with the manufacturer’s recommendations.

### 4.2. Cell Preparation and Analyses

#### 4.2.1. Jurkat Cells Permeabilization and Viability Measurements

Jurkat cells (#TIB-152, ATCC, Manassas, VA, USA) were grown at 37 °C and 5% CO_2_ and passaged before reaching a density of ~10^6^ cells/mL in RPMI 1640 medium (Sigma–Aldrich) supplemented with 10% fetal bovine serum (FBS, Atlanta Biologicals, Minneapolis, MN, USA), 1% penicillin/streptomycin (PS), 1.5 g/L sodium bicarbonate, 4.5 g/L D-(+) glucose, 10 mM HEPES, 1 mM sodium pyruvate, and 2 mM l-glutamine. The cells were counted on the day of experimentation with a bright-line cytometer (Hausser Scientific, Horsham, PA, USA), pelleted by centrifugation, and washed twice with 1 mL of HBSS (Sigma–Aldrich, without added calcium chloride, magnesium sulfate, sodium bicarbonate, or phenol red). The cells were resuspended in HBSS containing PI (1.5 µM final concentration), from which we placed 100 µL in wells (four wells/experimental sample) of tissue culture treated plates (Fisher Scientific). The fluorescence spectroscopy assessment was performed on Jurkat cells that were only exposed to PI (control), Jurkat cells exposed to PI and lysenin (10 nM final concentration), and Jurkat cells exposed to PI and lysenin, but with the channels being blocked by the addition of chitosan (25 nM final concentration in the samples) 20 min. after fluorescence recording started. This amount of lysenin was much greater than what we used for the planar lipid membrane experiments, and it was required to achieve a significant permeabilization of a very high number of cell membranes within a short time frame (i.e., ~20 min.). The total surface area of the cells that underwent permeabilization was much larger than the surface area of the bilayer membrane and, consequently, the lysenin’s concentration was greater. For the permeabilization experiments, the time evolution of the PI’s fluorescence was monitored every five minutes at room temperature with a Biotek Synergy MX microplate reader (Biotek, Winooski, VT, USA) that was set for 535 nm excitation, 617 nm emission, and a sensitivity of 65 units. The fluorescence of all samples was measured for a total duration of 50 min.

The viability of Jurkat cells was assessed by employing the Alamar Blue (resazurin) method [42]. For this test, we seeded 0.2 mL of ~5 × 10^5^ cells/mL into flat-bottom 96-well plates in HBSS, rested the samples for 30 min. in an incubator (37 °C, 5% CO_2_), treated them with chitosan (up to 125 nM final concentration), and continued the incubation for 20 h. After the addition of 10% v/v resazurin (Sigma–Aldrich), the samples were incubated for an additional four hours. The reduction of blue, non-fluorescent resazurin to fluorescent resorufin in viable cells was measured with the microplate reader (530 nm excitation, and 590 nm emission wavelengths) and the viability reported as percent relative to control samples not treated with chitosan. The viability test was similarly performed on Jurkat cells that were exposed to 10 nM lysenin, with and without the addition of chitosan (25 nM final concentration).

#### 4.2.2. ATDC5 Cells Preparation and Experiments

ATDC5 cells (#99072806, Sigma–Aldrich, kindly supplied by the Biomolecular Research Center at Boise State University) were grown in 75 cm^2^ flasks (VWR International, Radnor, PA, USA) containing 15 mL of DMEM/F12 (Gibco Dulbecco’s modified medium—nutrient mixture F12, supplemented with 5% FBS and 1% PS, ThermoFisher Scientific). After achieving 70–80% confluency, the cells were trypsinized for 7 min. with 0.25% trypsin-EDTA (Sigma–Aldrich), followed by enzyme deactivation by the addition of 7 mL medium. The cells were counted with a bench hemocytometer, centrifuged, washed, and resuspended in fresh medium before re-seeding into 75 cm^2^ tissue-culture treated flasks. The cells were grown and maintained in an incubator at 37 °C and 5% CO_2_.

We monitored and characterized the permeabilization of cells with confocal microscopy. Glass-bottom (35 mm) culture dishes (MatTek, Ashland, MA, USA) were seeded with 2 mL of cell solution (~10^5^ cells/mL) and then incubated at 37 °C and 5% CO_2_ for 24 h. An AF546-Phal stock solution was prepared by mixing 300 units with 1.5 mL methanol, and a working solution was prepared by diluting 50 µL stock solution in 1 mL HBSS. The cells that were grown in glass-bottom dishes for 24 h were washed twice with 1mL of HBSS, followed by the addition of 100 µL of the same buffer. Each well was incubated with lysenin (10 nM final concentration) for ten minutes to induce permeabilization, washed twice with HBSS to remove the free lysenin, incubated with 100 µL of AF546-Phal for ten min., and then rinsed before imaging to reduce background signal. The control samples were prepared similarly, but without lysenin addition. Images were acquired with a Zeiss LSM 510 Meta confocal system paired with the Zeiss Axiovert Observer Z1 inverted microscope (Carl Zeiss, Inc., Thornwood, NY, USA), equipped with Ar 488 and HeNe 543 lasers, a band-pass emission filter (500–550 nm, green channel), and a long-pass emission filter (red channel). The collected images were processed with the ZEN 2009 imaging software (Carl Zeiss, Inc., Thornwood, NY, USA).

The experiments aimed at estimating ATDC5 cells viability were conducted similarly, but the cells were incubated with 2 mL DMEM media (to block the lysenin channels) for 5 h at 37 °C and 5% CO_2_, washed, exposed to 100 µL of 2 µM calcein-AM, and then imaged by confocal microscopy for combined permeabilization/viability assessments.

### 4.3. Liposome Preparation and Analysis

Liposomes were prepared by the extrusion method [53]. Chloroform solutions of 8 mg Aso, 2.5 mg SM, and 2.5 mg Chol were vacuum-dried overnight in a glass vial and the resulting lipid cake was slowly hydrated at 65 °C for five hours in 1 mL solution containing 135 mM KCl and 20 mM HEPES (pH 7.2). After each hour, the mixture was subjected to a freeze-thaw cycle by placing it in the freezer for twenty minutes, immediately followed by the immersion of the vial in hot water. Large aggregates that were visually observed in the solution after hydration were fragmented by short sonication (< 10 s) in a benchtop sonicator. After the completion of hydration, the lipid solution containing multilamellar liposomes was extruded 40 times through stacked polycarbonate filters (1 µm pore diameter, Avanti Polar Lipids) that were mounted in a Mini-Extruder (Avanti Polar Lipids) placed on a hotplate and kept at constant temperature (72 °C). After extrusion, the liposomes were cooled at room temperature and stored in a refrigerator for further experiments. A stock solution of calcein (50 µM) was prepared by dissolving it in 135 mM KCl, 20 mM HEPES, pH 7.2. Calcein fluorescence quenching by multivalent metal cations (Al^3+^, prepared as 10 mM solution in water from a stock AlCl_3_ solution—Fisher Scientific) was analyzed using a Fluoromax 4P fluorometer (Horiba Scientific, Piscataway, NJ, USA) set in emission mode (λ_ex_ = 485 nm, λ_em_ = 490 nm–550 nm). The fluorescence spectrum of 50 µM calcein solution (prepared in the KCl buffer) was recorded before and after successive addition of AlCl_3_ solution to the sample in the cuvette under continuous stirring. The quenching curve was plotted as relative fluorescence intensity at 520 nm versus [Al^3+^].

Image recording and analysis was performed with a Nikon TS100 microscope (Nikon Instruments Inc., Melville, NY, USA) in transmission/epifluorescence mode, which was equipped with an Infinity camera (Lumenera, Ottawa, Canada) and appropriate filters. After lysenin addition, trivalent metal ion removal was performed by precipitation upon the addition of a sodium phosphate solution [39] that was obtained by mixing the monobasic dihydrogen phosphate and dibasic monohydrogen phosphate forms.
